# Supplementary material for: Palmitoleoylethanolamide Is an Efficient Anti-Obesity Endogenous Compound: Comparison with Oleylethanolamide in Diet-Induced Obesity
Source: Nutrients. 2021 Jul 28;13(8):2589. doi: 10.3390/nu13082589 (PMC8400335; doi:10.3390/nu13082589)

Supplementary material

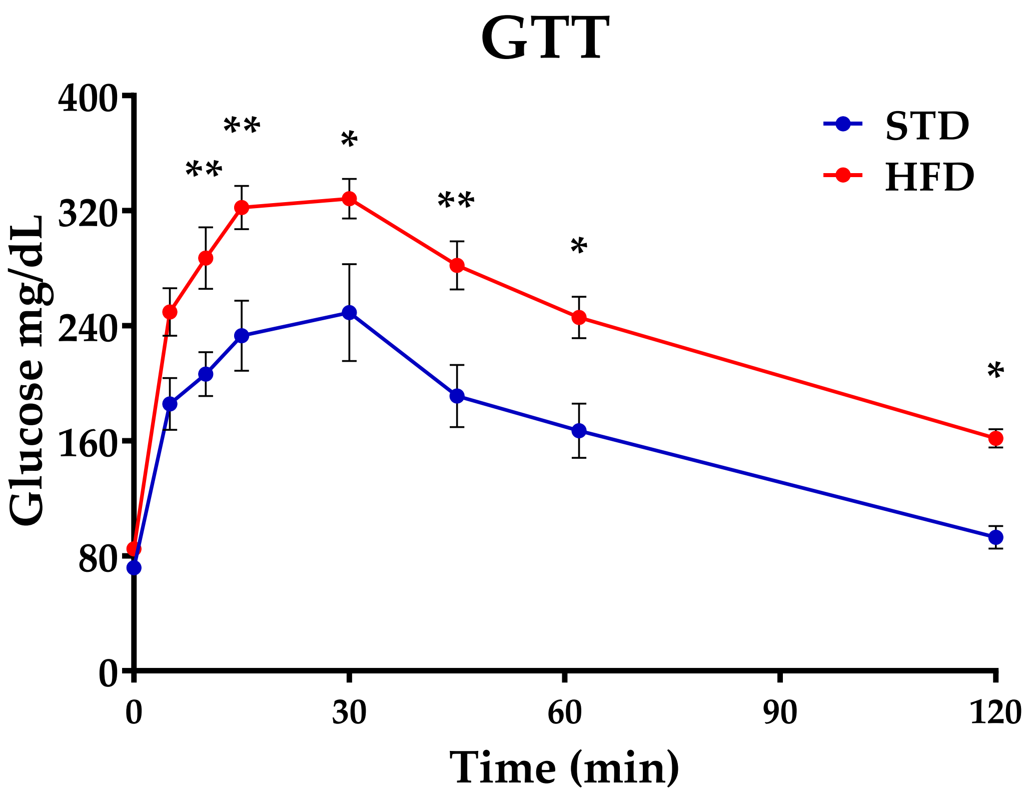

Figure S1. Glucose tolerant test (GTT) after 91 days of STD and HFD fed on Sprague Dawley rats. Animals were fasted for 18 h before they received an i.p. injection of glucose (2 g/Kg i.p.) Blood glucose concentrations were measured in blood drawn from the tail vein using a glucometer (AccuCheck, Roche, Germany), at 0 (basal level), 5, 10, 15, 30, 45, 60 and 120 min after the glucose injection. Points in the line are expressed as mean ± SEM (n = 10 animals/group). Two-way ANOVA and Bonferroni post-hoc test: (*) $p < 0.05$ and (**) $p < 0.01$ vs STD group

Figure S2: Two membranes were used for γ-Adaptin, FAS, SCD1 and FAAH immunoblotting (unedited blots).

Whole membranes 1 and 3. Red Ponceau Staining and individual membranes are shown below. FAS, SCD1 and FAAH bands are represented in Figure 8. All bands were quantified for histograms charts and statistical analysis, although only representative bands were shown in Figure 8. All proteins were normalized with its respective γ-Adaptin. Linoleoyl ethanolamide (LEA) was not included in the present study.

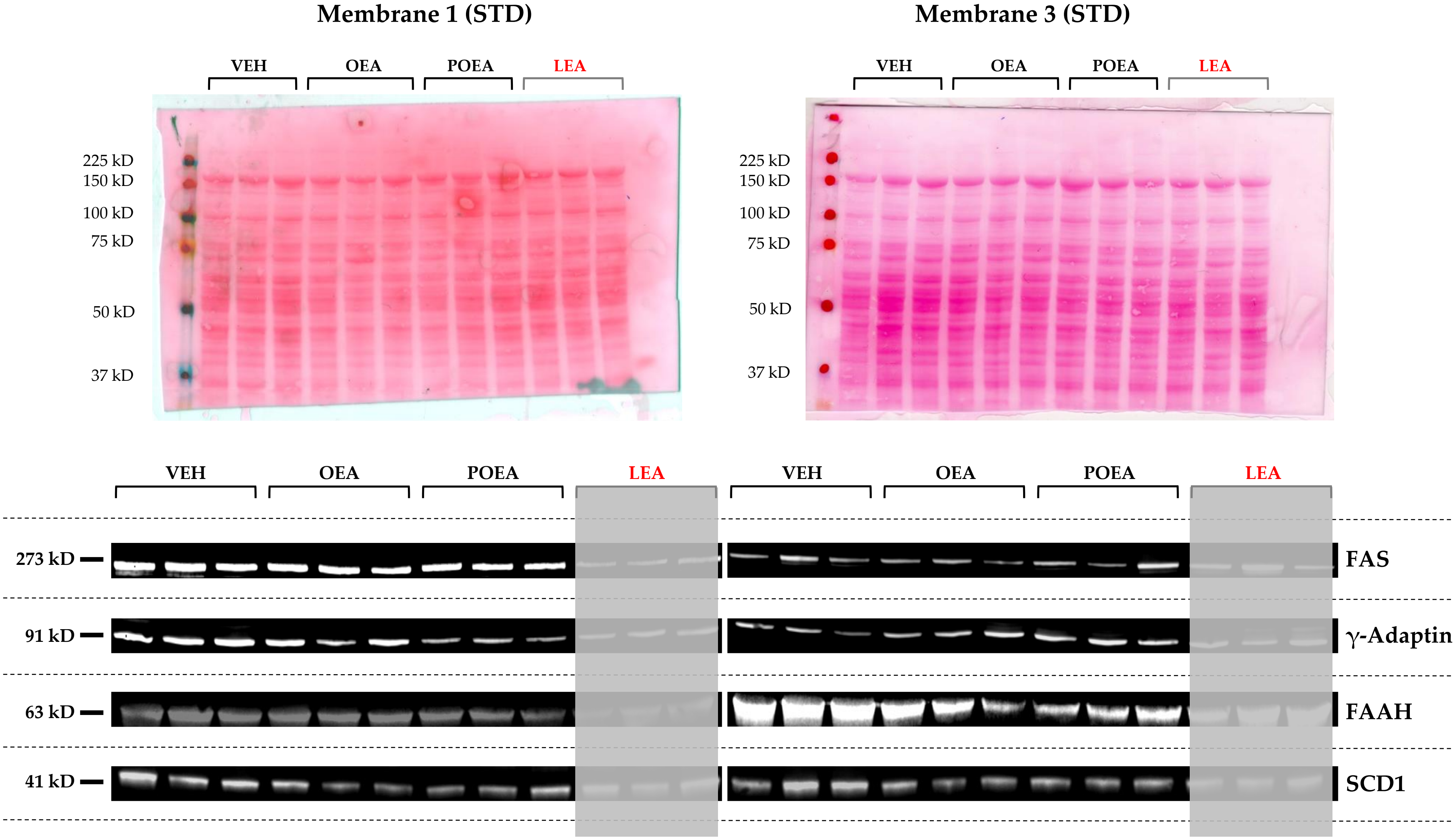

Figure S3: Two membranes were used for γ-Adaptin, FAS, SCD1 and FAAH immunoblotting (unedited blots).

Whole membranes 2 and 4. Red Ponceau Staining and individual membranes are shown below. FAS, SCD1 and FAAH bands are represented in Figure 9. All bands were quantified for histograms charts and statistical analysis, although only representative bands were shown in Figure 9. All proteins were normalized with its respective γ-Adaptin. Linoleoyl ethanolamide (LEA) was not included in the present study.

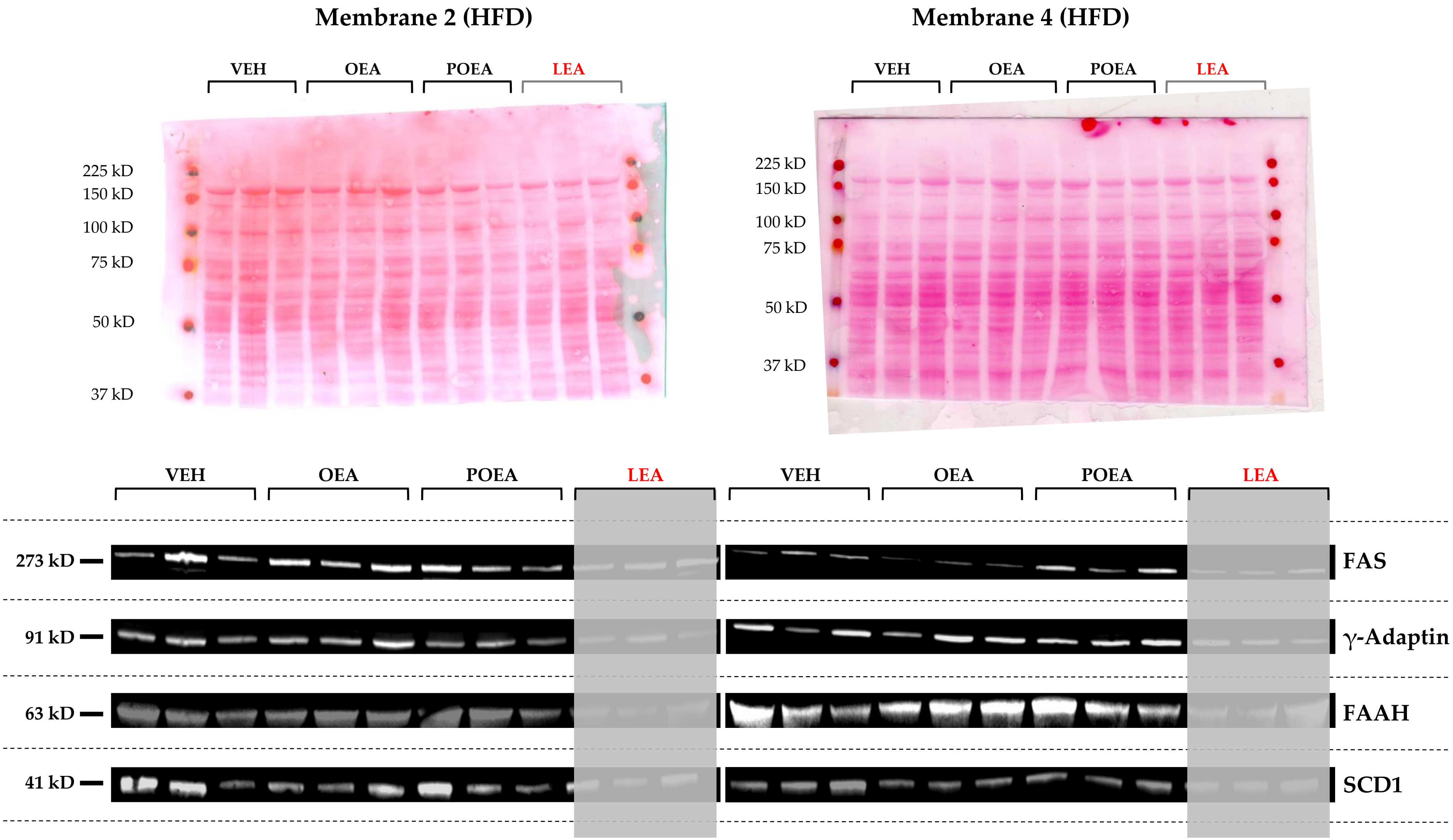

Supplement: Supplementary file 1 [file nutrients-13-02589-s001.zip › nutrients-1255455-supplementary.pdf]
